# Features of the CD1 gene family in rodents and the uniqueness of the immune system of naked mole-rat

**DOI:** 10.1186/s13062-024-00503-z

**Published:** 2024-07-29

**Authors:** Konstantin V. Gunbin, Gelina S. Kopeina, Boris Zhivotovsky, Alexey V. Zamaraev

**Affiliations:** 1 Center for Mitochondrial Functional Genomics, Immanuel Kant Baltic Federal University, Kaliningrad, 236016 Russia; 2 Institute of Molecular and Cellular Biology SB RAS, Novosibirsk, 630090 Russia; 3 Engelhardt Institute of Molecular Biology, RAS, Moscow, 119991 Russia; 4 Faculty of Medicine, MV Lomonosov Moscow State University, Moscow, 119991 Russia; 5 Division of Toxicology, Institute of Environmental Medicine, Karolinska Institutet, Box 210, Stockholm, 17177 Sweden

**Keywords:** Cluster of differentiation 1, CD1, Naked mole-rat, NKT cells

## Abstract

Cluster of Differentiation 1 (CD1) proteins are widely expressed throughout jawed vertebrates and present lipid antigens to specific CD1-restricted T lymphocytes. CD1 molecules play an important role in immune defense with the presence or absence of particular CD1 proteins frequently associated with the functional characteristics of the immune system. Here, we show the evolution of CD1 proteins in the Rodentia family and the diversity among its members. Based on the analysis of CD1 protein-coding regions in rodent genomes and the reconstruction of protein structures, we found that *Heterocephalus glaber* represents a unique member of the suborder Hystricomorpha with significant changes in protein sequences and structures of the CD1 family. Multiple lines of evidence point to the absence of CD1d and CD1e and probably a dysfunctional CD1b protein in *Heterocephalus glaber*. In addition, the impact of CD1d loss on the CD1d/Natural killer T (NKT) cell axis in the naked mole-rat and its potential implications for immune system function are discussed in detail.

## Background

**Fig. 1:**
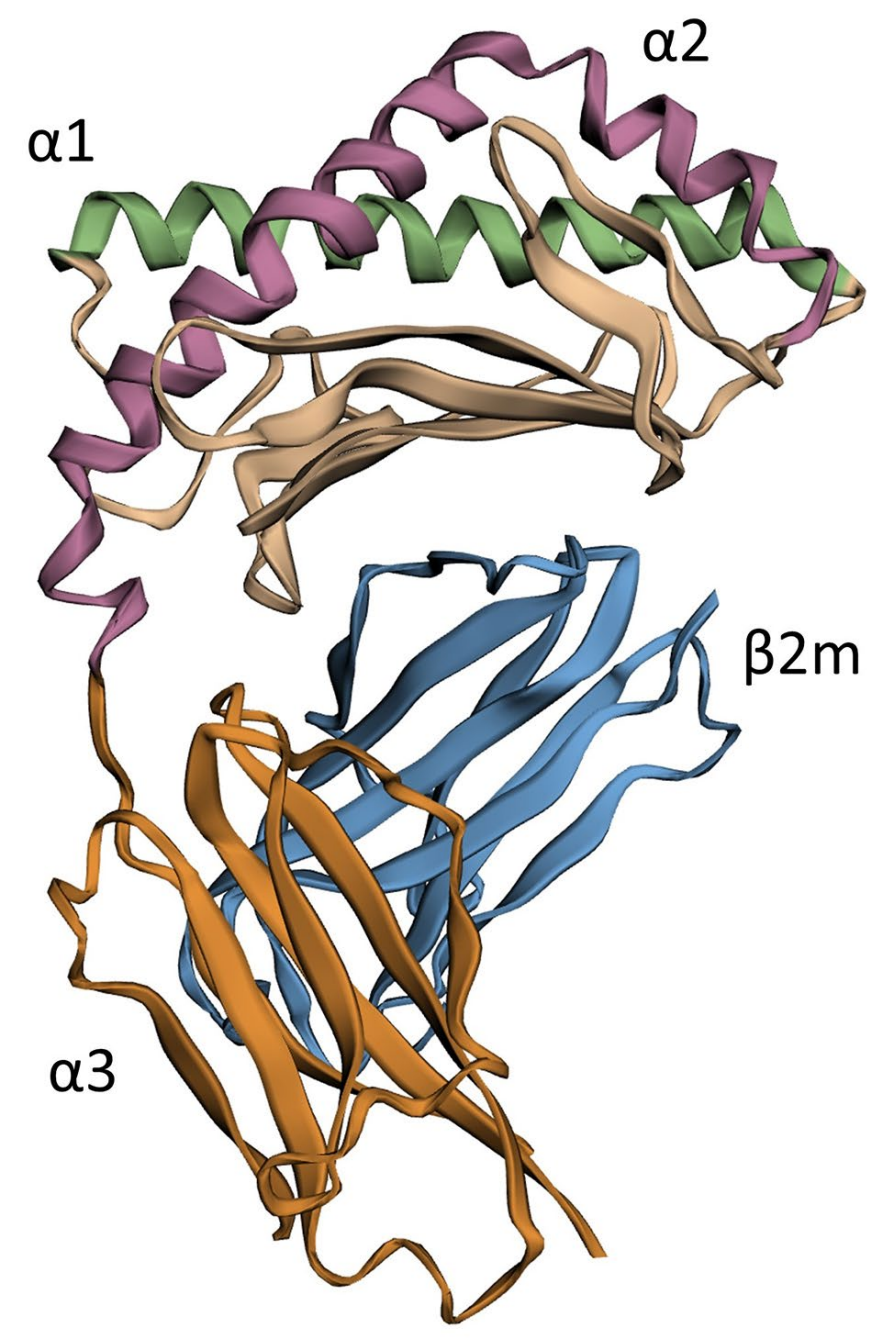
Domain structure of mouse CD1d1 protein. The heavy chain is composed of α1, α2, and α3 domains which are non-covalently associated with β-microglobulin (pdb:6mss). The antigen-binding hydrophobic cleft is formed by α1 + α2 domains. The α3 domain attaches CD1 molecules to membranes through a transmembrane segment

The cellular adaptive immune system is highly dependent on antigen-presenting molecules on the surface of cells and their interactions with T cells. The key members of antigen-presenting molecules in mammals is the family of major histocompatibility complex (MHC). The typical MHC class I and class II proteins are highly polymorphic and present pathogen or self-protein fragments to CD8^+^ and CD4^+^ T cells, respectively. However, some T cells could recognize non-peptide antigens, presented by MHC-like molecules, namely, Cluster of Differentiation or CD1 [1]. These proteins are non-polymorphic and expressed in many jawed vertebrates. Members of the CD1 family present self- and foreign lipid antigens to specific CD1-restricted T lymphocytes. The structure of CD1 proteins is similar to MHC I molecules, consisting of three extracellular domains (α1- α3) and non-covalently associated with β2-microglobulin (β2m) protein (Fig. 1). The MHC-like fold of CD1 makes possible the formation of a hydrophobic binding pocket that accommodates lipid-based antigens [2]. However, each type of CD1 protein is distinguished from the others by different intracellular trafficking, expression patterns on immune or epithelial cells, and, importantly, unique functions. Today, five members of the CD1 family are well recognized and based on sequence homology, classified into three groups: group I (CD1a, CD1b, and CD1c), group II (CD1d), and group III (CD1e) [3] (Supplementary Fig. 1).

Group I proteins are predominantly expressed on antigen-presenting cells to expose the diverse self and pathogen lipid antigens. T cells reactive to CD1-presenting lipid antigens mediate immune recognition and cytokine secretion. In contrast to group I, CD1d is widely expressed in different hematopoietic and non-hematopoietic cells, including epithelial cells [4]. A variety of glycolipids, phospholipids, lipopeptides, or non-lipid small molecules from bacteria, fungi, pollen, or self-antigens could be presented by CD1d molecules to the natural killer T (NKT) cells [4, 5] which have both immune-activating and immunosuppressive roles. The third group of the CD1 family members, which is represented by the soluble intracellular protein CD1e, is mainly located in the Golgi apparatus in dendritic cells and thymocytes. In mature dendritic cells, CD1e molecules localize in endosomal and lysosomal compartments, where they facilitate the presentation of endogenous and exogenous lipid antigens by CD1 proteins [6].

CD1 antigen presentation is an ancient system of immune response that is found in mammals, birds, and reptiles, but absent in fish [7]. The number of CD1 genes and the composition of CD1 loci vary widely among mammalian species. Some species have survived without one of the five CD1 gene types, the other species consist of 14 CD1 genes, but no mammalian species lacking all CD1 proteins has been identified. Therefore, the CD1 system plays an indispensable role in immune response and the presence or absence of several CD1 proteins could be associated with the positive selection by evolutionary forces and/or pathogenic environment of mammalian species. To date, most preclinical studies have been conducted on members of the Rodentia order, such as mice, rats, and guinea pigs [8]. However, even within a suborder, the presence of different sets of CD1 genes varies greatly between species. Within the Rodentia order, the mouse genome has only two CD1d genes (CD1d1 and CD1d2) [9], while the rat genome has only one CD1d gene [10]. Guinea pigs were initially reported to have four functional CD1b genes, three CD1c genes, one CD1e gene, and five pseudogenes [11]. Subsequent studies have shown that guinea pigs have functional CD1d gene [12]. Therefore, the composition of the CD1 locus has only been accurately described for two model species among the entire diversity of rodents. Here, we present data concerning the evolution of CD1 proteins in the Rodentia order and the diversity across CD1 members and propose the functional relationship between these proteins and the immune response.

## Methods

**Fig. 2:**
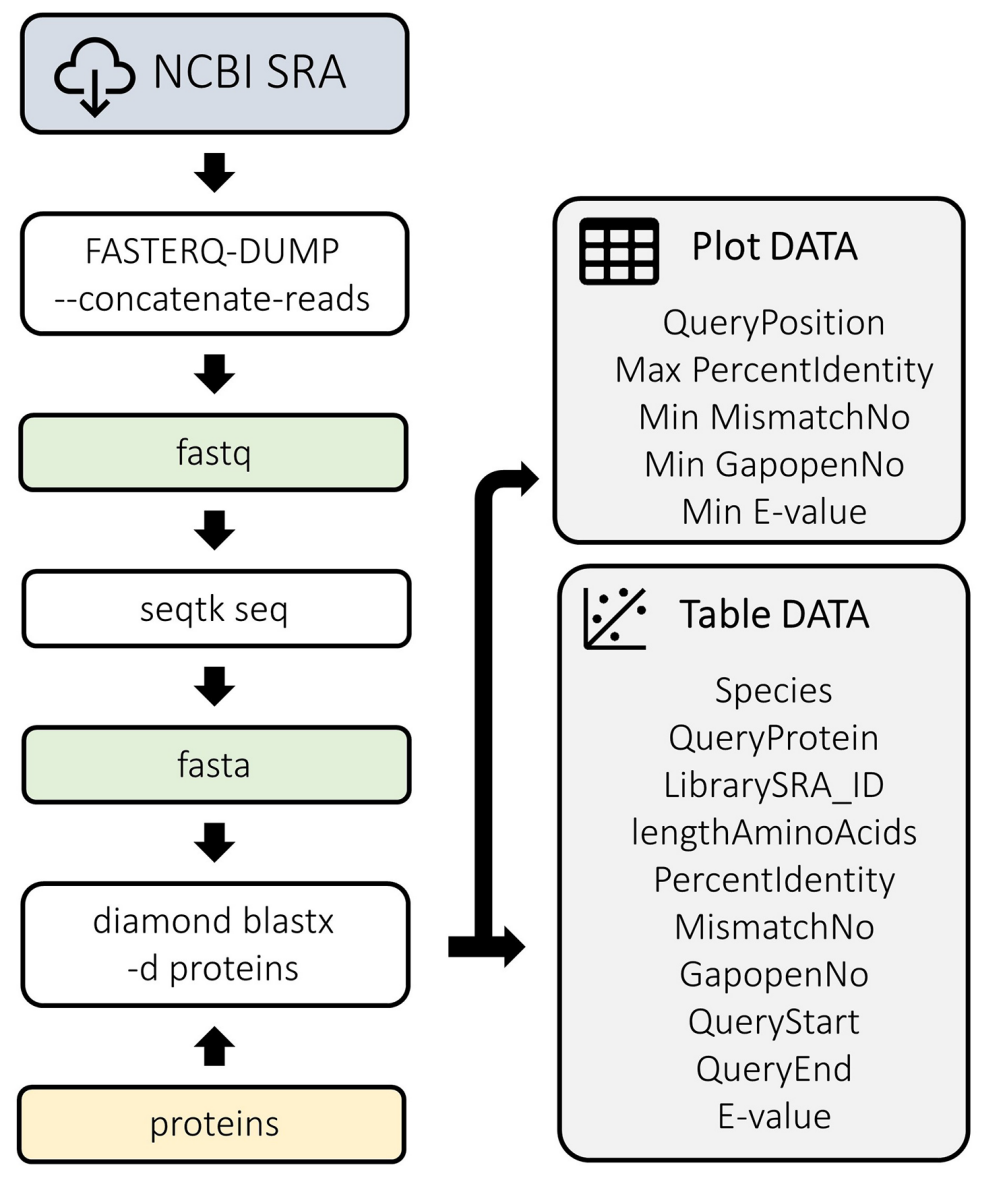
A simplified scheme of the core of the first analysis step, the identification of any possible CD1 protein-coding regions (and protein-coding regions for key proteins in CD1-related pathways) in several rodent genomes even if these genomes are not carefully assembled yet (or have the preliminary genome release)

A pipeline has been implemented in this study (Fig. 2). The pipeline can be divided into four distinct steps. (1) Whole genome data libraries were obtained from NCBI SRA using SRA tools through parallel data downloading with fasterq-dump. (2) Fastq raw reads data were then converted and filtered into a multi-fasta format using the seqtk tool. (3) Homologous reads to query protein sequences were searched for in parallel using the diamond software. (4) Results were summarized in text tables and graphic plots, including identification of best-matched reads against query proteins based on the percent of target identity, number of mismatches and gaps in the target, and E-value.

### Obtaining whole genome data

We downloaded genomic data from NCBI SRA and searched CD1 proteins into the 8 sequencing projects (s.p.) of *C. canadensis*, 8 s.p. of *C. porcellus*, 123 s.p. of *C. griseus*, 14 s.p. of *D. ordii*, 16 s.p. of *F. damarensis*, 145 s.p. of *H. glaber*, 72 s.p. of *I. tridecemlineatus*, 23 s.p. of *J. jaculus*, 35 s.p. of *M. monax*, 31 s.p. of *M. unguiculatus*, 30 s.p. of *M. auratus*, 6 s.p. of *M. agrestis*, 22 s.p. of *N. galili*, 25 s.p. of *O. princeps*, 25 s.p. of *O. degus*, 71 s.p. of *O. cuniculus*, 43 s.p. of *S. dauricus* and 5 s.p. of *U. parryii*. We used annotated query of CD1 proteins (and protein-coding regions for key proteins in CD1 related pathways) from *Mus musculus* and *Rattus norvegicus* as well as from *C. porcellus* despite preliminary genome assembly status (at the moment of sequence retrieval).

### Parallel searching reads homologous to query protein sequences

Protein-to-DNA sequence homology was searched by diamond tool v.0.9.30.131 using default options. All query proteins (and corresponding DNA coding sequences) and the whole set of diamond result tables (containing diamond sequence retrieval hits), as well as Perl scripts for analysis, are shown as a single bundle Supplementary archive #1. Tables summarizing best diamond hits (reads most similar to query) for each query protein positions (4 values computed: percent of best-hit identity, number of mismatches per best hit, gap open per best hit, and the lowest E-value) for each species under analysis as well as Perl scripts are shown as Supplementary archive #2.

In order to reconstruct proteins based on the intersection of diamond hits (reads most similar to query) a simple pipeline in Perl language was implemented, which performs read extraction, filtering, and translation based on diamond result tables: one for reads with tolerable similarity to query (E-value < 1E-4 and percent of identity > 40) and another for reads with low similarity to query (30 < percent of identity ≤ 40). Extraction of translatable regions in reads was perform by standard samtools faidx command for all 3 translation frames taking into consideration strand information from diamond result tables. The DNA-to-protein translation was done by transeq tool of EMBOSS software package v.6.6.0. All Perl scripts, as well as the resulting set of reads, are shown in Supplementary archive #3. The final step of sequences analyses, protein reconstruction based on the intersection of best diamond hit reads, implemented as separate Perl script recProtein.pl. We show this script as a part of Supplementary archive #4 which additionally contains other supplementary Perl scripts as well as reconstructed CD1 proteins for all species under analysis and their residue-per-residue descriptions (percent of best hit identity, number of mismatches per best hit, gap open per best hit and the lowest E-value).

## Reconstructing of CD1 protein by AlphaFold2

After reconstructing of CD1 protein sequences for 7 species (*F. damarensis. H. glaber*,* M. unguiculatus*,* M. musculus*,* O. princeps*,* O. cuniculus*,* C. porcellus*) reconstruction of 3D protein structure was performed using web available AlphaFold Colab Python notebook (https://colab.research.google.com/github/deepmind/alphafold/blob/main/notebooks/AlphaFold.ipynb*).* Since we aimed to reconstruct the structure of CD1 monomers (and extrapolate protein functionality based on these predictions) we did not use Colab Pro. The raw results of AlphaFold2 protein structure predictions (both raw json Predicted Aligned Errors and pdb protein structures) and graphical representations of 3D structures, Predicted Aligned Errors, and predicted LDDT are shown in Supplementary archive #5. Additionally, to be sure that the AlphaFold Colab predictions agree with AlphaFold2 predictions the proteomes of AlphaFold Protein Structure Database (https://console.cloud.google.com/marketplace/product/bigquery-public-data/deepmind-alphafold) were used for these species for searching CD1 protein homologs (if there exists). No significant disagreements in predicted protein structures were found.

### Sequence alignment and phylogenetic analysis

Amino acid sequence alignments and phylogenetic tree reconstruction were produced based on simple ClustalO software using the Uniprot portal (https://www.uniprot.org). The genomic sequence comparison between species was performed based on default Ensembl tools (https://www.ensembl.org).

### Mapping of RNA-seq SRA project reads homologous to Ensembl query protein sequences

A simple diamond database was constructed, comprising four possible CD1d proteins (ENSHGLP00000039071, ENSHGLP00000040141, ENSHGLP00000057523, ENSHGLP00000057161) from the preliminary genome assembly of *Heterocephalus glaber* (GCA_944319715.1), accessible via the Ensembl portal (Ensembl Release 111, January 2024). As previously described in the section entitled “Parallel searching reads homologous to query protein sequences”, the protein-to-DNA search was conducted using the diamond tool.

## Results

Two steps of bioinformatics analysis have been carried out: (1) retrieving sequences to identify potential CD1 protein-coding regions in various rodent genomes and (2) roughly estimating of possible protein functionality based on structural data if such a protein-coding region was identified. The NCBI SRA was used as a data source for sequence retrieval in the following rodent species from the main suborders: Castorimorpha (*Castor Canadensis*,* Dipodomys ordii*), Hystricomorpha (*Cavia porcellus*,* Fukomys damarensis*,* Heterocephalus glaber*,* Octodon degus*), Myomorpha (*Cricetulus griseus*,* Jaculus jaculus*,* Meriones unguiculatus*,* Mesocricetus auratus*,* Microtus agrestis*,* Nannospalax galili*), Sciuromorpha (*Ictidomys tridecemlineatus*,* Marmota monax*,* Spermophilus dauricus*,* Urocitellus parryii*) and Lagomorpha *(Ochotona princeps*,* Oryctolagus cuniculus)* as an outgroup.

**Fig. 3:**
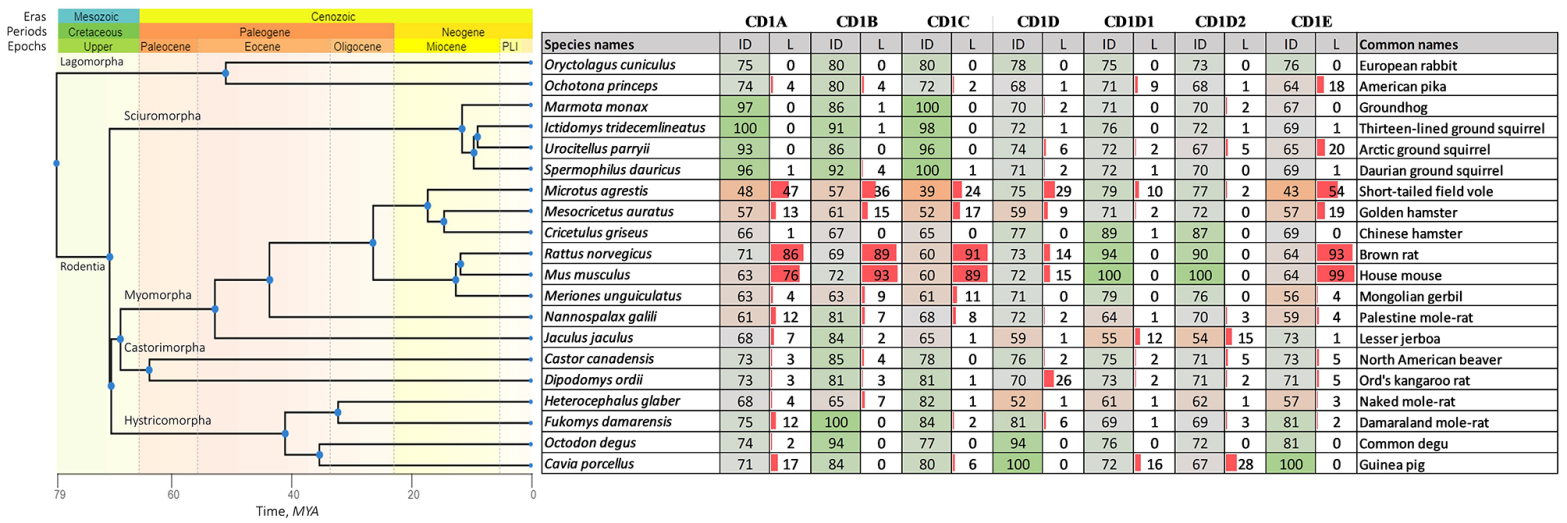
The medians of best query-target sequence similarities i.e., percent of identity to query sequence (ID columns) for CD1a, CD1b, CD1c, CD1d, CD1d1, CD1d2, CD1e genes and the percentage of position loss (L columns) for these genes. PLI is an abbreviation for Pliocene. MYA is an abbreviation for Million Years Ago. Molecular time estimates and tree topology are taken from the TimeTree 5 database [14]. The suborders of the Rodentia order, including Sciuromorpha, Castorimorpha, Hystricomorpha, and Myomorpha, are indicated in the TimeTree

To obtain the best query-target sequence similarity scores for each query position the best-matched read was identified (Supplementary archive #2). These scores were gathered from similarity datasets between species-specific reads from libraries (target) listed in Supplementary archive #1 and the following protein queries: CD1d1 & CD1d2 proteins of *Mus musculus*, CD1b protein of *Fukomys damarensis*, CD1a & CD1c proteins of *Marmota marmota*, CD1d & CD1e proteins of *Cavia porcellus*. The choice of these proteins as queries was based on their high level of annotation within the Rodenta taxon. In the analysis, we included the CD1d1 and CD1d2 genes that were described in *Mus musculus* due to the inability to differentiate between them using the similarity-based methodology and the different roles of CD1d1 and CD1d2 molecules in the mouse thymus [13]. To directly match reads with proteins the Diamond software was used (see Methods). The presence or absence of CD1 genes was estimated by calculating the percentage of identity to the query sequence and the percentage of position loss (Fig. 3). If the percentage of identity is below 60–70 or the percentage of position loss is greater than 20–30, it could indicate the potential loss of a functional gene.

We have shown that different CD1 proteins are specific for different Rodent species and generally tend to reduce in a clade. Specifically, *Mus musculus* and *Rattus norvegicus* possess exclusively CD1d1 and/or CD1d2 proteins. *Microtus agrestis* and *Mesocricetus auratus* are likely to have only full-length CD1d1 and/or CD1d2 proteins, while *Meriones unguiculatus* and *Cricetulus griseus* could potentially retain other full-length genes from the CD1 family (Fig. 3). It is presumed that *Jaculus jaculus* and *Nannospalax galili* possess a CD1b protein. Interestingly, *Jaculus jaculus*, which emerged during the Paleogene period, displays the least sequence-similarity of CD1d, CD1d1, and CD1d2 within the Myomorpha suborder and may have lost the CD1d gene (Fig. 3). Within the suborders of Castorimorpha, Sciuromorpha, and Hystricomorpha, it is probable that the majority of the scrutinized species possess all genes of the CD1 family as shown by high sequence-similarity scores and the absence of position losses. Nevertheless, *Heterocephalus glaber*, also known as naked mole-rat, exhibits considerably low sequence-similarity scores for both CD1d and CD1e genes (Fig. 3). Additionally, the lengths of normalized translated peptides without stop-codons were calculated by multiplying the maximum peptide length (from analyzed read without stop-codons) by the fraction of read identity to the query protein (calculated after translating the read into protein). The distribution of normalized translated peptide (from retrieved reads) lengths without stop-codons for reconstructed CD1d, CD1d1, CD1d2, and CD1e in *Heterocephalus glaber* are significantly lower (*p*-value < 0.01; U-test) than those for CD1a, CD1b, and CD1c, indicating the absence of functional CD1d and CD1e genes (Supplementary Fig. 2).

**Fig. 4:**
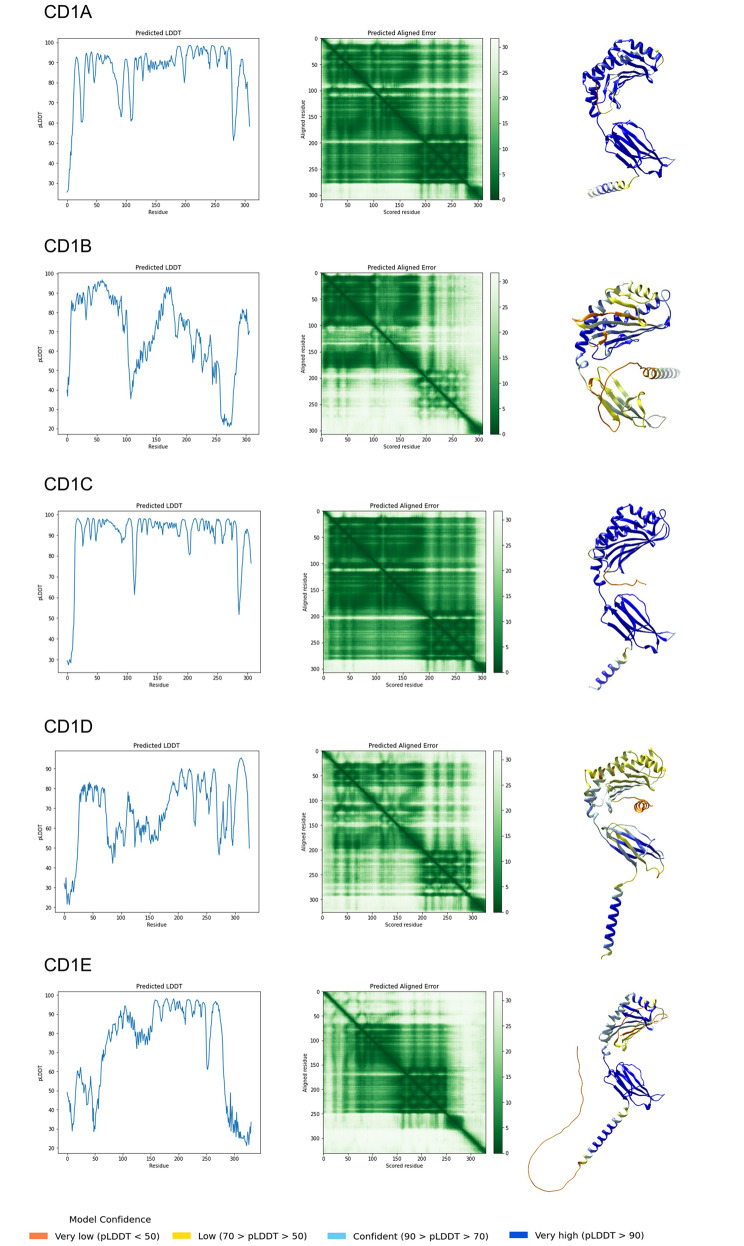
The Alphafold prediction for the 3D structures of the CD1 proteins in *Heterocephalus glaber.* The pLDDT (predicted Local Distance Difference Test) and PAE (Predicted Aligned Error) plots provide information on structure confidence. If the protein regions are classified as α-helix or β-sheet and have pLDDT scores below 70 (yellow or orange), the CD1 fold structure cannot be deemed reliable. Therefore, the same functionality cannot be considered in this case. The PAE score is defined as the expected positional error at residue X, measured in Ångströms (Å), if the predicted and actual structures were aligned on residue Y. A dark green corresponds to a low PAE, which indicates a high reliability of the relative position of the residues. Conversely, a lighter color corresponds to a lower confidence

To more accurately analyze the presence or absence of CD1 functions protein primary sequences were reconstructed based on the best-matched reads (matched to our query protein sequences). The protein sequences were employed to generate protein structures using the AlphaFold technique (refer to the Methods section) for *Heterocephalus glaber*. Additionally, the study examined model organisms, namely *Fukomys damarensis* and *Cavia porcellus*, from the Hystricomorpha suborder that are neighboring the naked mole-rat. The analysis also encompassed *Jaculus jaculus*, which has seemingly lost its CD1d genes, alongside the model species *Mus musculus* and the out-group species *Ochotona princeps* and *Oryctolagus cuniculus* (Supplementary archive #5). The reliability of different regions of the predicted structure was identified using AlphaFold confidence measures, specifically pLDDT (predicted Local Distance Difference Test) and PAE (Predicted Aligned Error). Our analysis confirmed the absence of functional structures of CD1d and CD1e proteins in *Heterocephalus glaber* based on low or very low pLDDT scores and high PAE scores in α1 and α2 domains (Fig. 4 and Supplementary Fig. 3). Furthermore, the existence of an unstructured region in the α1 and α3 domains of the CD1b structure may result in a defective or non-functional protein (Fig. 4 and Supplementary archive #6). *Fukomys damarensis*, *Ochotona princeps*, and *Oryctolagus cuniculus* possess all spectra of CD1 proteins with a high probability, as indicated by high pLDDT scores. The analysis of pLDDT and PAE plots of *Jaculus jaculus* confirmed the lack of a functional CD1d gene (Supplementary archive #5). It should be noted that *Cavia porcellus* lacks a reliable indication of CD1a protein (information about *Cavia porcellus* CD1a protein has been removed by the UniProt consortium and CD1a functional expression is uncertain, as indicated in [15]). In contrast, *Mus musculus* only displays a distinct signal for the existence of CD1d1 and CD1d2 proteins.

**Fig. 5:**
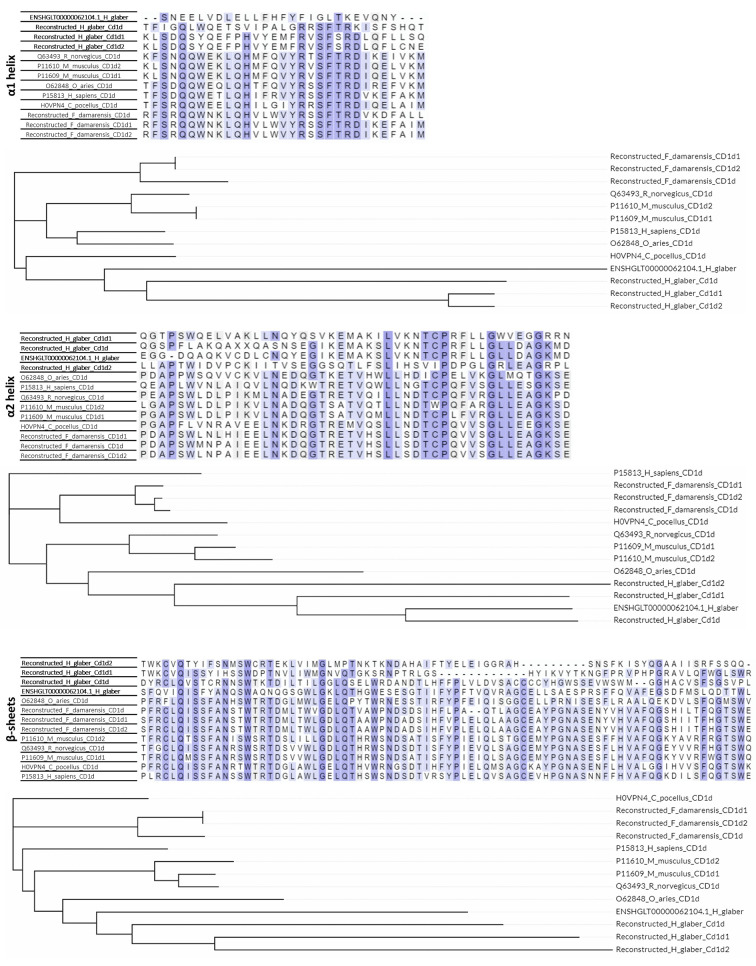
The multiple sequence alignments and phylogenetic trees of the α1 and α2 helixes and the β-sheets in the antigen-presenting part of CD1d protein sequences. The Uniprot ID is indicated for known protein sequences. The amino acid sequences were aligned with the ClustalO software, and a neighbor-joining tree was generated

**Fig. 6:**
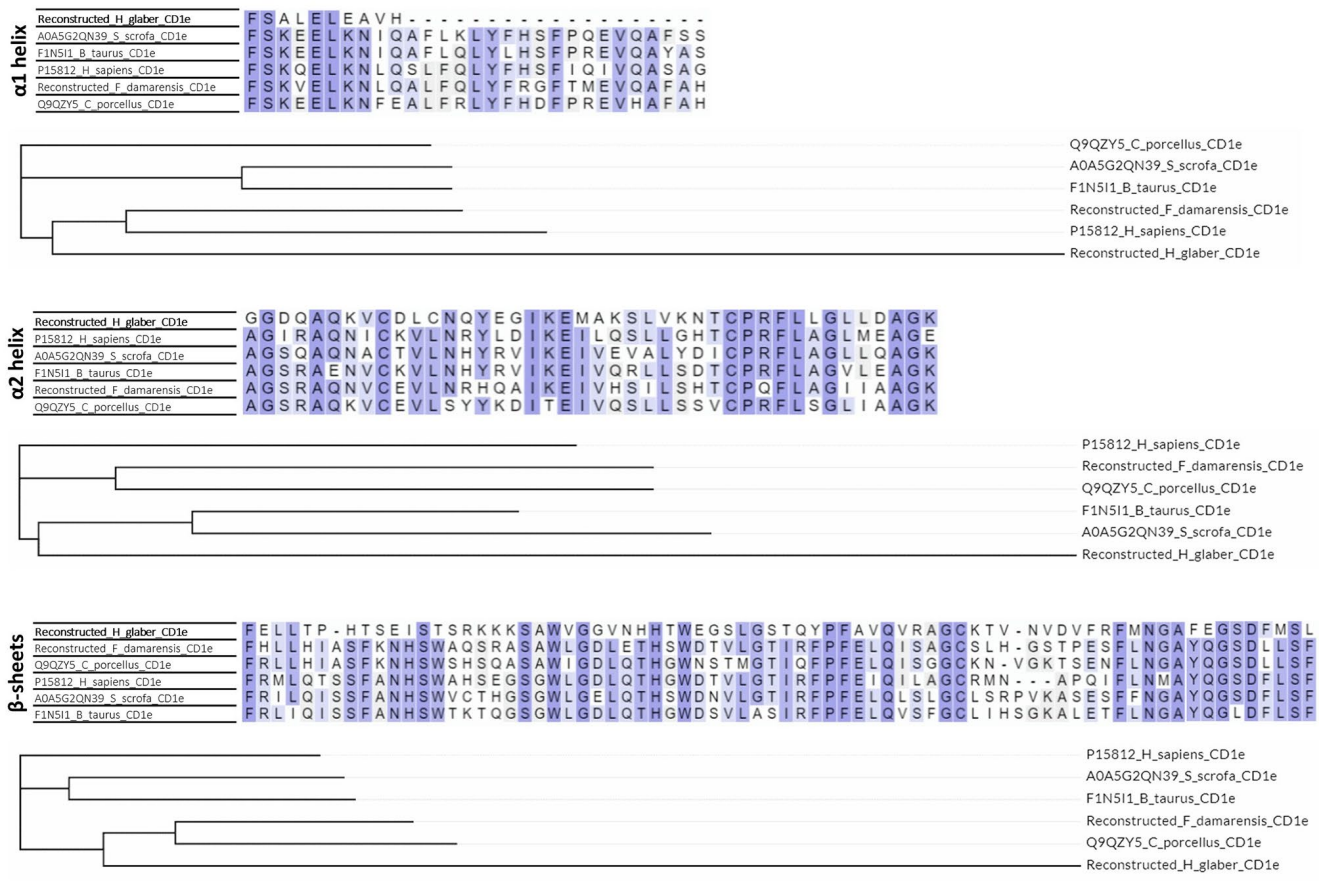
The multiple sequence alignments and phylogenetic trees of the α1 and α2 helixes and the β-sheets in the antigen-presenting part of CD1e protein sequences. The Uniprot ID is indicated for known protein sequences. The amino acid sequences were aligned with the ClustalO software, and a neighbor-joining tree was generated

Furthermore, the α1 and α2 helixes and the β-sheets which represent the antigen-presenting part of the CD1 molecules were separately analyzed (α1 and α2 domains in Fig. 1). A considerable number of substitutions were observed in these regions in CD1d and CD1e in *Heterocephalus glaber* in comparison with the same domains of humans, mice, guinea pigs, and neighboring species (Figs. 5 and 6). The identity of the α helices and β-sheets of the reconstructed CD1d, CD1d1, and CD1d2 in *Heterocephalus glaber* was found to be in the range of 20–40% when compared to other species with known protein sequences of model species (Supplementary Table 1). Furthermore, the majority of the crucial residues across the length of the antigen-binding cleft, which are involved in the TCRs interaction [16] have been substituted in the reconstructed CD1d sequences (Supplementary Table 2) in the naked mole-rat, which precludes the TCR interaction. The identity of the α helixes of the reconstructed CD1e in *Heterocephalus glaber* to other species with known protein sequence was in the range of 40–50%, while the identity of the β-sheets was 34–38% (Supplementary Table 1). The dataset also includes α1 and α2 helixes and the β-sheets regions from Ensembl canonical transcript ENSHGLT00000062104.1 from preliminary assemble genome (GCA_944319715.1) from one female naked mole-rat that mapped on the mouse chromosome 3:86892244–86,898,703 location which contains the CD1d2 gene (Fig. 5). Nevertheless, the length of branches in phylogenetic trees corresponding to the *Heterocephalus glaber* was significantly longer than any others, indicating a high number of substitutions in these regions (Figs. 5 and 6, phylogenetic trees). The genome data allows for the analysis of interspecies variability. Notably, the branch lengths leading to these sequences are longer than those leading to different families (human, sheep, mice, etc.). This evidence clearly indicates that functional and structural conservation are not attributed to this locus.

Moreover, an analysis of the primary genome assembly of the naked mole-rat (GCA_944319715.1), accessible via the Ensembl portal (Ensembl Release 111, Jan-2024), was conducted to ascertain whether the *Heterocephalus glaber* genome region exhibits similarity with mouse CD1d1 and CD1d2 transcripts. The *Heterocephalus glaber* genome region, which may exhibit CD1d homology when compared to the CD1d1 and CD1d2 paralogs of *Mus musculus*, is characterized by the presence of frameshifts and substantial structural alterations (Supplementary archive #7). To identify the expression of any potential transcripts from the naked mole-rat genome (GCA_944319715.1) that map to the mouse CD1d gene locations, the comprehensive bulk RNA-seq experiments of *Heterocephalus glaber* tissues (BioProject PRJNA770623 (bone marrow, cervical and thoracic thymus, peripheral blood, lymph node) [17], PRJNA826455 (skin), PRJNA144103 (liver) [18]) were analyzed. However, no significant sequence similarity to Ensembl transcripts was identified in the RNA-seq data (tables summarizing the best reads similarity to queries (greater than 90%) are presented in Supplementary archive #8).

Thus, the family of CD1 proteins is highly divergent in the Rodentia order, particularly in the Myomorpha suborder. In contrast, the Sciuromorpha, Castorimorpha, and Hystricomorpha suborders are more conservative and likely have most variants of CD1 molecules. In this context, *Heterocephalus glaber* represents a unique member of the suborder Hystricomorpha, which has undergone the loss of functional CD1 genes that could potentially impact the functionality of the immune system.

## Discussion

Computational analysis performed by us revealed high evolutionary diversity in the rodent CD1 protein family, which plays a crucial role in non-peptide antigen presentation and immune response. The genome sequence analysis and reconstruction of CD1 proteins allowed us to distinguish the specific rodent group based on the sequence homology of CD1 members and associate it with the functional features of the rodent immune system.

We demonstrated that the suborders Hystricomorpha (guinea pig, naked mole-rat, and others) and Sciuromorpha (squirrel, woodchuck, and others) represent the most conservative and evolutionary old group of Rodents, which contains almost all types of CD1 genes. The Myomorpha suborder is the most divergent and evolutionarily novel rodent group. The species of the Dipodoidae clade, such as *Nannospalax*,* Jaculus*, locate closer to the root of the evolution tree and demonstrate a greater degree of CD1 genes homology to other rodent clades, while the species of the Muroidea clade, including *Mus musculus* and *Rattus Norvegicus*, contain only one member of the CD1 family – duplicated CD1d gene (CD1d1, CD1d2) or single CD1d.

Despite the high conservatism of the Hystricomorpha suborder, we identified that *Heterocephalus glaber*, also known as the naked mole-rat, is a unique animal that contains CD1c and CD1a genes, and seemingly CD1b gene unlike a neighboring member - *Fukomys damarensis* and other species in the Hystricomorpha suborder. Among all rodents, only a few species, such as *Heterocephalus glaber* and *Jaculus jaculus*, lack the CD1d gene. However, no detailed immunological studies have yet been conducted on the immune system of *Jaculus jaculus*.

Recent detailed studies of the naked mole-rat described the unique features of their immune system which are characterized by the absence of canonical natural killer (NK) cells that are tightly involved in the antiviral response and a high myeloid-to lymphoid cell ratio. Moreover, the presence of the NKT cell population in the naked mole-rat is also not described [19, 20]. Considering the indispensable role of CD1d in the development of NKT cells [21], it appears that the CD1d/NKT axis has been lost in the immune system of the naked mole-rat during evolution, possibly due to its atypical mammalian behavior.

It is important to note that the experiments with CD1d knockout mice demonstrate the crucial requirement of this protein for the development of NKT cells and the antiviral response [21]. The CD1d knockout mice have diminished B cell responses and reduced numbers of IL-4-secreting cells during influenza and vaccinia virus infection [22]. In response to the herpesvirus infection, these knockout mice exhibited a significantly reduced ability to clear the viral load, as well as decreased levels of IFN- γ and IL-12 [23]. Moreover, herpes simplex virus 1 has been shown to specifically inhibit CD1d-mediated antigen presentation by suppressing the recycling of CD1d on the cell surface and NKT cell activation, thereby, enhancing viral pathogenicity [24, 25]. It is worth noting that experiments with herpesvirus vectors resulted in a lethal outcome for all naked mole-rats [26]. Additionally, it was shown that inbreeding in the naked mole-rat is associated with increased susceptibility to coronavirus infection [27]. Seemingly, the high susceptibility of naked mole-rats to viruses, particularly to herpesvirus, may be partially attributed to the loss of the CD1d/NKT cell axis.

Another fascinating immune feature, that was described in recent research, is the failure to induce psoriatic skin inflammation upon application of imiquimod (IMQ) [28]. Imiquimod is a toll-like receptor (TLR) 7 and 8 agonist that triggers inflammatory skin reactions that closely resemble those seen in psoriasis. Kisipan ML et al. revealed that the skin of IMQ-treated naked mole-rat did not demonstrate any pathological changes. However, in mice, IMQ can induce inflamed skin lesions resembling plaque-type psoriasis [29]. In psoriatic lesions, NK receptor-positive cells, including NK and NKT cells, play a crucial role in cytokine release [30]. CD1d proteins are diffusely expressed on keratinocytes and the CD1d - NKT cell interaction has been shown to be important for IFN-γ production [31, 32]. In turn, NK cells are increased in psoriatic skin lesions and involved in proinflammatory cytokine secretion (TNF-α, IFN-γ), induction of adhesion molecules, and chemokine expression in keratinocytes [30]. Thus, the lack of NK cells and the CD1d/NKT-axis in the naked mole-rat could be a valid reason for the resistance to psoriatic skin inflammation.

The comparative study of naked mole-rat and mouse immunity revealed another intriguing feature of innate immune defense mechanisms. The proportion of splenic macrophages, their phagocytic capability, and cytokine production were significantly greater in the naked mole-rat than in the mouse spleen [33]. Further investigation identified that most macrophages of the naked mole-rat in a naïve state express NK1.1, a surface antigen typically present in NK cells and NKT cells in mice. This suggests that the macrophage could function as an NK cell [34]. Additionally, recent studies have demonstrated that the naked mole-rat has a large population of γδT cells with an NK-like cytotoxic effector phenotype [20, 35]. Thus, it can be postulated that the immune system of the naked mole-rat could partially compensate for the loss of NKT and NK cells by stimulating myeloid cells, which is consistent with a high myeloid-to-lymphoid cell ratio, and a high prevalence of NK-like γδT cells, that successfully copes with the elimination of senescent or damaged cells to extend the naked mole-rat’s life span.

## Data Availability

No datasets were generated or analysed during the current study.

## Declarations

### Supporting information

All Supplemental archives and figures have been deposited in the following repository: 10.6084/m9.figshare.25601145.

## Abbreviations

CD1: cluster of differentiation 1
IFN-γ: interferon gamma
IL-12: interleukin 12
MHC: major histocompatibility complex
NK: natural killer cells
NKT: natural killer T cells
PAE: predicted aligned error
pLDDT: predicted local distance difference test
TCR: T cell receptor
TLR: toll-like receptor
